# Exploring Tractography: An Analysis of Brain Connectivity Patterns in Men

**DOI:** 10.7759/cureus.73991

**Published:** 2024-11-19

**Authors:** Bhamini Sharma, Amit Mittal, Rahul Sharma, Pinki Rai, Kirandeep K Aulakh

**Affiliations:** 1 Anatomy, Maharishi Markandeshwar Institute of Medical Science &amp; Research, Ambala, IND; 2 Radiodiagnosis, Maharishi Markandeshwar Institute of Medical Science &amp; Research, Ambala, IND; 3 Anatomy, All India Institute of Medical Sciences, Rishikesh, IND; 4 Anatomy, Government Medical College & Hospital, Chandigarh, IND

**Keywords:** brain mri normal, diffusion tensor tractography (dtt), fractional anisotropy (fa), mean diffusivity (md), white matter changes on mri

## Abstract

Introduction: White matter tracts that connect different parts of the brain comprise the structural connectome, which is essential to its operation. Assessing behavioral changes and brain health requires an understanding of these tracts. Diffusion tensor imaging (DTI), in particular, allows for the thorough viewing and characterization of these routes in tractography. In order to assess the impact of aging on white matter integrity, this study examines the 10 main white matter tracts in men, paying particular attention to volume, fractional anisotropy (FA), and mean diffusivity (MD).

Materials and methods: A cohort of 49 men aged 18-50 years was examined using a Philips Multiva 1.5T MRI. DTI scans were performed after obtaining informed consent. Participants with neuronal disorders were excluded. Ten tracts were assessed: inferior fronto-occipital fasciculus (IFOF), superior fronto-occipital fasciculus (SFOF), inferior longitudinal fasciculus (ILF), superior longitudinal fasciculus (SLF), cingulum, corticospinal tract (CST), forceps major, forceps minor, uncinate fasciculus, and anterior thalamic radiation (ATR). Statistical analysis employed Kruskal-Wallis tests to compare age groups.

Results: Significant age-related differences were observed in the IFOF, which exhibited notable changes in both volume and MD. Specifically, the IFOF's volume peaked in the 31-40 age group (14.42 ± 6.05) and declined in the 41-50 age group (8.71 ± 5.07), with a statistically significant p-value of 0.019. In parallel, MD increased significantly with age, moving from 0.86 ± 0.08 in the 18-30 group to 1.09 ± 0.13 in the 31-40 group and stabilizing at 0.96 ± 0.12 in the 41-50 group (p < 0.001). Notably, while the FA values remained relatively stable across age groups (p = 0.063), the increase in MD suggests a decline in neural efficiency or potential myelin degradation. Other tracts, including the SFOF and SLF, displayed stability in volume, FA, and MD across age groups, indicating a degree of resilience in certain neural pathways.

Conclusion: This study highlights the utility of tractography in understanding age-related changes in white matter, such as in Alzheimer’s disease, age- and sex-related abnormalities, and dementia, particularly emphasizing the IFOF's sensitivity. Findings offer insights into brain connectivity and neurological health, indicating a need for further contribution to inform interventions aimed at cognitive preservation.

## Introduction

The brain is a multi-networked organ that is interconnected and performs intricate and dynamic neurocognitive activities [1]. The interactions between geographically confined regions are responsible for its functioning [1]. A link between cortical, subcortical, and cerebellar areas forms the brain's structural connectome, which allows different neural messages to pass along white matter. Because white matter tracts in humans affect behavioral changes and account for population variance, studying them is therefore fascinating [2]. To comprehend the functioning of the brain in both healthy and diseased states, one must be able to recognize and describe the nerve fiber tracts that link different functional regions. Tractography reconstructs the white matter fibers, estimating the local orientation of nerve fibers voxel by voxel, which enables to see the paths of particular bundles of white matter fibers, and it may be possible to quantify the characteristics of tracts [3]. This offers intriguing possibilities to evaluate the effects of illnesses on certain tracts. A tract's size may be determined once its location has been established [3].

For instance, Gongora et al. explored white matter tracts in a Cuban sample, examining the relationships between tract metrics and factors such as age, cerebral hemispheres, and gender [4]. Additionally, Mormina et al. conducted MRI tractography on the corticospinal tract and arcuate fasciculus in patients with high-grade gliomas, providing both qualitative and quantitative insights [5]. Moreover, Fortin et al. utilized diffusion tensor imaging (DTI) to investigate the white matter tracts of a healthy individual, contributing to our understanding of functional connectivity [6].

The aim of this study is to characterize 10 major white matter tracts in men, focusing on volume, fractional anisotropy (FA), and mean diffusivity (MD) derived from DTI scans. By analyzing these parameters, we aim to enhance our understanding of how age and other factors influence white matter integrity in this demographic.

## Materials and methods

Study design

This descriptive observational study was conducted at the Department of Anatomy, MMIMSR, Mullana, Ambala, Haryana, India. This study was approved by the Research Ethics Committee with Project No. IEC-2206.

Inclusion and exclusion criteria

The study included 49 men aged 18-50 years. Only those subjects who were willing to participate were included in the study. Prior written and verbal consent for the study was taken from all the subjects, both in English and in vernacular. A total of 51 participants were excluded from the study on the basis of neurological disorder, brain tumor, and brain injury.

Data collection

The data were collected using a Philips Multiva 1.5T MRI machine in the Department of Radiology, MMIMSR, Mullana, Ambala, Haryana, India. DTI scans were taken of the subjects with prior written and verbal consent. The tracts included in the study were inferior fronto-occipital fasciculus (IFOF), superior fronto-occipital fasciculus (SFOF), inferior longitudinal fasciculus (ILF), superior longitudinal fasciculus (SLF), cingulum, corticospinal tract (CST), forceps major, forceps minor, uncinate fasciculus, and anterior thalamic radiation (ATR). The volume of each tract was estimated. FA and MD, which tell about fiber density, axonal diameter, and nerve cell orientation, were obtained.

Statistical analysis

In this study, three age groups were created: Group I (18-30 years), Group II (31-40 years), and Group III (41-50 years), and the data were entered in an Excel sheet. IBM SPSS Statistics for Windows, Version 28.0 (Released 2021; IBM Corp., Armonk, New York, United States) was used for statistical analysis. As the data were not normally distributed across these age categories, non-parametric tests (Kruskal-Wallis test) were used for group comparison. The P-value considered statistically significant was <0.05 in our study.

## Results

**Figure 1:**
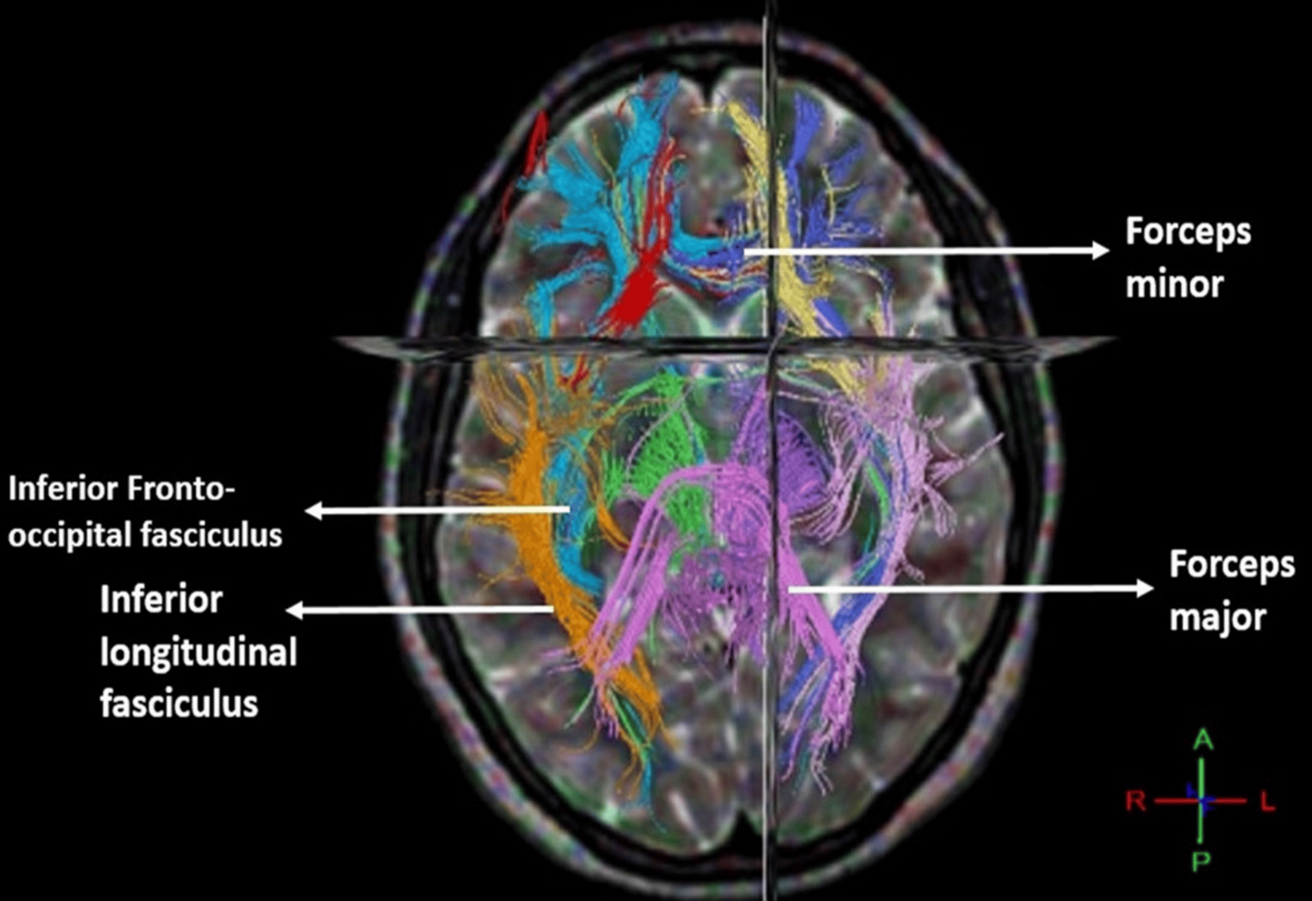
Tracts shown: IFOF, ILF, forceps major, and forceps minor in men. IFOF: inferior fronto-occipital fasciculus; ILF: inferior longitudinal fasciculus.

**Figure 2:**
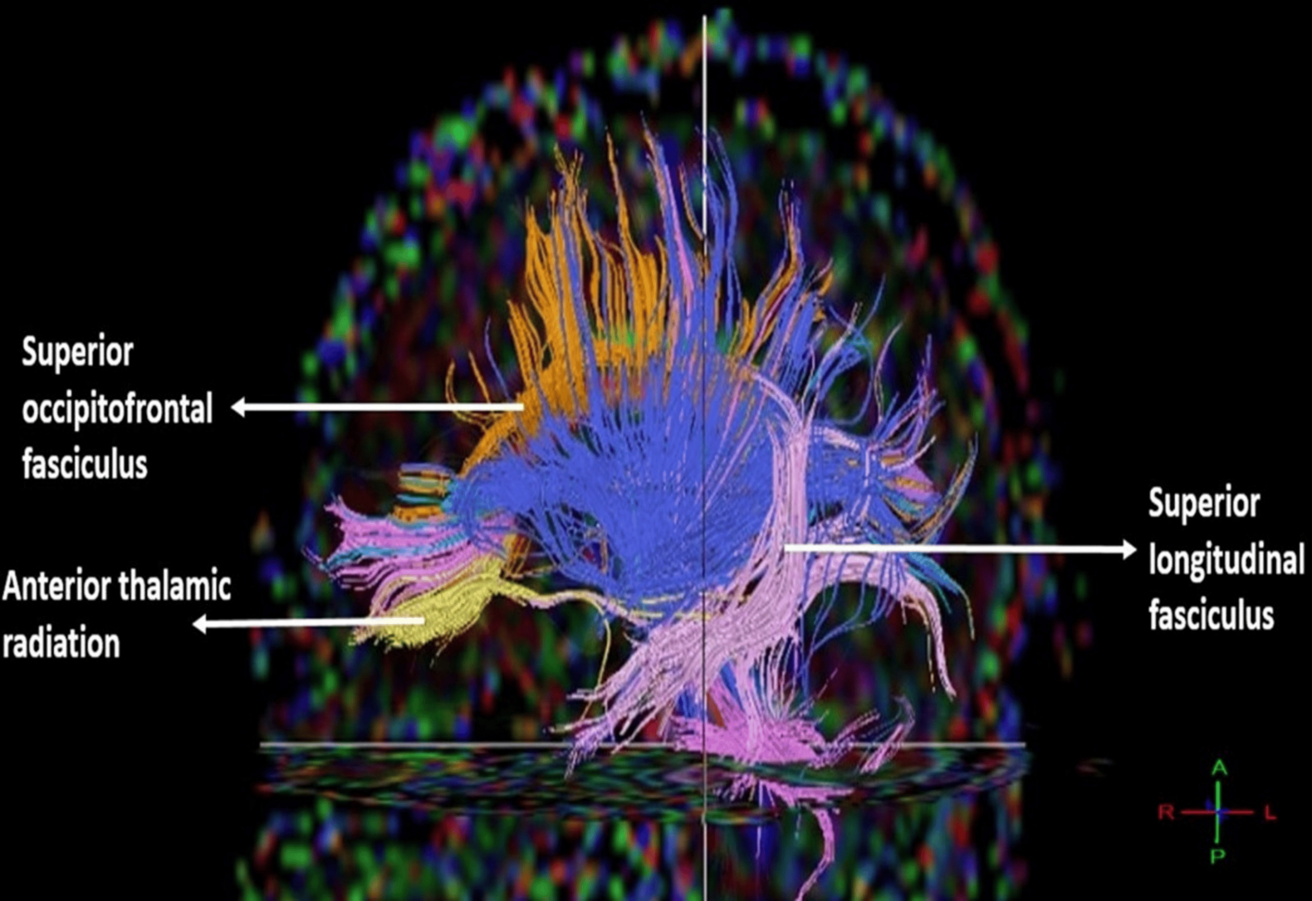
Tracts shown: SFOF, ATR, and SLF in men. SFOF: superior fronto-occipital fasciculus; SLF: superior longitudinal fasciculus; ATR: anterior thalamic radiation.

**Figure 3:**
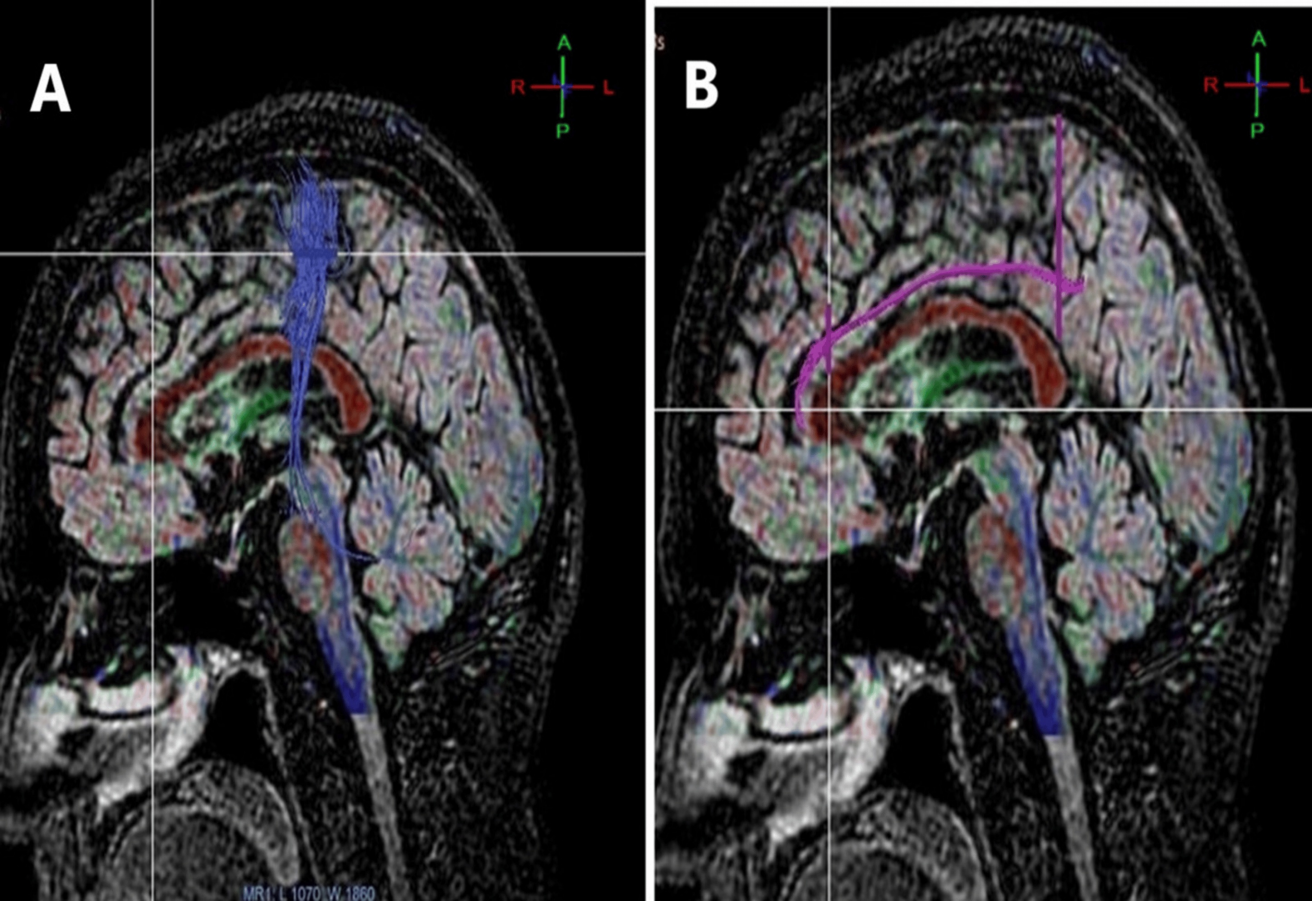
(A) CST; (B) cingulum in men. CST: corticospinal tract.

In our study, 49 men aged between 18 and 50 years were included. The sample was divided into three age groups: Group I (18-30 years, n = 15), Group II (31-40 years, n = 7), and Group III (41-50 years, n = 27). Table 1 presents the mean and standard deviation for the volume, FA, and MD of the 10 white matter tracts examined. Figures 1-3 depict the images of the tracts after the DTI scan of the brain.

**Table 1:** Mean and standard deviation for the volume, FA, and MD of the 10 white matter tracts examined in men aged 18-50 years. Non-parametric tests (Kruskal-Wallis test) and chi-square tests were used for group comparisons. The 10 white matter tracts are IFOF, SFOF, ILF, SLF, cingulum, CST, forceps major, forceps minor, uncinate fasciculus, and ATR. FA: fractional anisotropy; MD: mean diffusivity; IFOF: inferior fronto-occipital fasciculus; SFOF: superior fronto-occipital fasciculus; ILF: inferior longitudinal fasciculus; SLF: superior longitudinal fasciculus; CST: corticospinal tract; ATR: anterior thalamic radiation. ***P values of volume and MD of IFOF are significant.

Tracts	Parameters	Age			P-value	Chi-square (χ^2^)
		18-30 Years (n = 15)	31-40 Years (n = 7)	41-50 Years (n = 27)		
IFOF	Volume***	8.54 ± 7.20	14.42 ± 6.05	8.71 ± 5.07	0.019	7.957
	FA	0.50 ± 0.05	0.45 ± 0.03	0.48 ± 0.06	0.063	5.539
	MD***	0.86 ± 0.08	1.09 ± 0.13	0.96 ± 0.12	<0.001	16.482
SFOF	Volume	9.16 ± 6.15	12.06 ± 5.45	10.77 ± 8.26	0.291	2.47
	FA	0.50 ± 0.05	0.49 ± 0.05	0.50 ± 0.04	0.816	0.406
	MD	1.02 ± 0.18	0.94 ± 0.05	0.90 ± 0.09	0.085	4.941
SLF	Volume	8.57 ± 5.23	14.38 ± 8.52	11.46 ± 6.62	0.257	2.718
	FA	0.49 ± 0.03	0.48 ± 0.05	0.49 ± 0.04	0.989	0.021
	MD	0.88 ± 0.08	0.90 ± 0.08	0.93 ± 0.13	0.731	0.627
ILF	Volume	7.21 ± 4.93	8.68 ± 4.34	8.60 ± 4.83	0.494	1.412
	FA	0.49 ± 0.05	0.49 ± 0.04	0.47 ± 0.05	0.424	1.718
	MD	0.91 ± 0.10	0.94 ± 0.09	0.95 ± 0.13	0.560	1.161
Cingulum	Volume	6.85 ± 4.54	7.14 ± 7.61	5.03 ± 4.07	0.434	1.671
	FA	0.47 ± 0.06	0.49 ± 0.06	0.46 ± 0.06	0.561	1.157
	MD	0.94 ± 0.12	1.01 ± 0.20	0.94 ± 0.16	0.585	1.074
CST	Volume	5.97 ± 3.37	9.47 ± 8.75	6.89 ± 4.40	0.876	0.265
	FA	0.53 ± 0.05	0.52 ± 0.05	0.52 ± 0.05	0.584	1.076
	MD	0.85 ± 0.07	0.85 ± 0.08	0.89 ± 0.13	0.722	0.652
Forceps major	Volume	3.75 ± 4.08	3.51 ± 1.02	3.35 ± 3.07	0.513	1.333
	FA	0.50 ± 0.07	0.50 ± 0.12	0.46 ± 0.06	0.058	5.711
	MD	0.88 ± 0.08	0.90 ± 0.12	0.94 ± 0.14	0.633	0.913
Forceps minor	Volume	2.99 ± 1.78	3.20 ± 1.34	2.49 ± 1.55	0.394	1.861
	FA	0.50 ± 0.09	0.50 ± 0.07	0.48 ± 0.05	0.224	2.995
	MD	0.89 ± 0.17	0.90 ± 0.08	0.88 ± 0.09	0.538	1.24
Uncinate fasciculus	Volume	3.19 ± 1.76	2.66 ± 2.35	3.28 ± 1.96	0.658	0.837
	FA	0.46 ± 0.05	0.47 ± 0.05	0.46 ± 0.03	0.478	1.477
	MD	1.00 ± 0.21	0.88 ± 0.07	0.92 ± 0.12	0.411	1.777
ATR	Volume	2.62 ± 1.80	3.07 ± 1.99	2.89 ± 2.43	0.892	0.229
	FA	0.49 ± 0.06	0.48 ± 0.07	0.45 ± 0.06	0.133	4.035
	MD	0.88 ± 0.09	0.88 ± 0.15	0.88 ± 0.12	0.650	0.86

The IFOF stood out in this research as the only tract exhibiting significant changes related to age in both volume and MD. Specifically, the volume peaked in Group II (ages 31-40) and decreased in Group III (ages 41-50), while MD increased significantly with age. These patterns suggest that the IFOF may be particularly sensitive to aging, potentially reflecting changes in neural connectivity or integrity that warrant further exploration. The relatively stable FA values across the age groups imply that while volume and MD are affected, the directional diffusion of water molecules (an indicator of fiber integrity) remains consistent.

In contrast, the other tracts examined, such as the SFOF and SLF, did not show significant variations in volume, FA, or MD with age. This relative stability may suggest that these tracts are less susceptible to age-related changes.

## Discussion

This study employed tractography to analyze the white matter tracts in a sample of 49 men, focusing on key parameters: volume, FA, and MD. The findings highlight significant changes within IFOF, underscoring the importance of age as a critical factor influencing brain connectivity and structural integrity. The observed increase in MD with age, particularly in the IFOF, may signal a decline in neural efficiency or increased water diffusion in the extracellular space, potentially indicative of myelin degradation or axonal loss. Such changes could have functional repercussions, possibly impacting cognitive processes linked to the IFOF, such as visual processing and integration of sensory information. For instance, Gongora et al. explored white matter tracts in a Cuban sample, examining the relationships between tract metrics and factors such as age, cerebral hemispheres, and gender [4]. Additionally, Mormina et al. conducted MRI tractography on the corticospinal tract and arcuate fasciculus in patients with high-grade gliomas, providing both qualitative and quantitative insights [5]. Moreover, Fortin et al. utilized DTI to investigate the white matter tracts of a healthy individual, contributing to our understanding of functional connectivity [6]. The stability in other tracts suggests a degree of resilience in certain neural pathways as men age. Studies done by Sullivan ad Pfefferbaum [7] and Bennett and Madden [8] highlighted that the IFOF exhibited greater vulnerability compared to other tracts, with widespread decreases in FA and increases in MD across multiple white matter tracts in older adults, reinforcing that white matter deteriorates with aging, thus aligning with our study. Taki et al. [9], however, showed changes in SLF that are responsible for maintaining structural integrity better over time, which aligns with this body of research. Resnick et al. [10] conducted a longitudinal study on 92 nondemented older adults, demonstrating significant declines in white matter integrity over time. These findings can be compared to our study's cross-sectional results.

This study quantifies white matter tracts, providing insight into neurological and psychiatric disorders that exhibit gender- and age-related differences. For example, Alzheimer’s disease and dementia are often characterized by disrupted white matter integrity, particularly in the IFOF and cingulum [11]. In addition to offering insights into the structural foundations underlying individual variations in behavior and cognition, Yan et al. [12] implied that the anatomical network architecture in the human brain is related to sex and brain size. Dayan et al. [13] studied the optic radiation using DTI, highlighting its importance for neurosurgical planning in children and teens with uncontrollable epilepsy who are being evaluated for temporal lobectomy. Choi et al. [14] reviewed the human brain's greatest commissural tract, the corpus callosum, and linked structural differences to clinical, behavioral, cognitive, and functional age- and sex-related abnormalities.

Ogut et al. evaluated Guillain-Mollaret triangle (GMT) or myoclonic triangle in human participants, most of which used DTI, MRI, or a combination of both, illustrating that stroke, brainstem cavernous malformations, and structural abnormalities are among the several underlying causes of hypertrophic olivary degeneration (HOD), a typical outcome of GMT injury, according to the research [15]. The abovementioned studies are in alignment with our study. However, a small sample size is the limitation of our study.

## Conclusions

This study emphasizes the importance of tractography in characterization of the tracts and elucidating the relationship between age and white matter integrity in men. Findings contribute to the understanding of how aging affects brain connectivity and neurological health. The implications of these findings extend beyond basic neuroscience; they hold significance for understanding neurological disorders characterized by disrupted white matter integrity, such as Alzheimer’s disease, age- and sex-related abnormalities, and dementia. It gives us an insight into the anatomical network architecture in the human brain, establishing normative values for white matter tracts. It also helps us detect pathological changes in clinical contexts. Thus, this study highlights the significance of the use of tractography in visualizing the human brain and its implications for neuronal disorders.

## References

[1] Grover VP, Tognarelli JM, Crossey MM, Cox IJ, Taylor-Robinson SD, McPhail MJ (2015). Magnetic resonance imaging: principles and techniques: lessons for clinicians. J Clin Exp Hepatol.

[2] Carr HY, Purcell EM (1954). Effects of diffusion on free precession in nuclear magnetic resonance experiments. Phys Rev.

[3] Wakana S, Caprihan A, Panzenboeck MM (2007). Reproducibility of quantitative tractography methods applied to cerebral white matter. Neuroimage.

[4] Góngora D, Domínguez M, Bobes MA (2016). Characterization of ten white matter tracts in a representative sample of Cuban population. BMC Med Imaging.

[5] Mormina E, Longo M, Arrigo A (2015). MRI tractography of corticospinal tract and arcuate fasciculus in high-grade gliomas performed by constrained spherical deconvolution: qualitative and quantitative analysis. AJNR Am J Neuroradiol.

[6] Fortin D, Aubin-Lemay C, Boré A, Girard G, Houde JC, Whittingstall K, Descoteaux M (2012). Tractography in the study of the human brain: a neurosurgical perspective. Can J Neurol Sci.

[7] Sullivan EV, Pfefferbaum A (2005). Neurocircuitry in alcoholism: a substrate of disruption and repair. Psychopharmacology (Berl).

[8] Bennett IJ, Madden DJ (2014). Disconnected aging: cerebral white matter integrity and age-related differences in cognition. Neuroscience.

[9] Taki Y, Kinomura S, Sato K, Goto R, Wu K, Kawashima R, Fukuda H (2011). Correlation between gray/white matter volume and cognition in healthy elderly people. Brain Cogn.

[10] Resnick SM, Pham DL, Kraut MA, Zonderman AB, Davatzikos C (2003). Longitudinal magnetic resonance imaging studies of older adults: a shrinking brain. J Neurosci.

[11] Hasan KM, Iftikhar A, Kamali A (2009). Development and aging of the healthy human brain uncinate fasciculus across the lifespan using diffusion tensor tractography. Brain Res.

[12] Yan C, Gong G, Wang J (2011). Sex- and brain size-related small-world structural cortical networks in young adults: a DTI tractography study. Cereb Cortex.

[13] Dayan M, Munoz M, Jentschke S (2015). Optic radiation structure and anatomy in the normally developing brain determined using diffusion MRI and tractography. Brain Struct Funct.

[14] Choi CH, Lee JM, Koo BB, Park JS, Kim DS, Kwon JS, Kim IY (2010). Sex differences in the temporal lobe white matter and the corpus callosum: a diffusion tensor tractography study. Neuroreport.

[15] Ogut E, Armagan K, Tufekci D (2023). The Guillain-Mollaret triangle: a key player in motor coordination and control with implications for neurological disorders. Neurosurg Rev.

